# Is there a causal relationship between stress and migraine? Current evidence and implications for management

**DOI:** 10.1186/s10194-021-01369-6

**Published:** 2021-12-20

**Authors:** Anker Stubberud, Dawn C. Buse, Espen Saxhaug Kristoffersen, Mattias Linde, Erling Tronvik

**Affiliations:** 1 Department of Neuromedicine and Movement Sciences, NTNU Norwegian University of Science and Technology, Trondheim, Norway; 2 National Advisory Unit on Headaches, Department of Neurology, St. Olavs Hospital, Trondheim, Norway; 3 Department of Neurology, Albert Einstein College of Medicine, Bronx, NY USA; 4 Department of Neurology, Akershus University Hospital, Lørenskog, Norway; 5 Department of General Practice, University of Oslo, Oslo, Norway

**Keywords:** Headache, Daily hassles, Trigger, Behavioral treatment

## Abstract

**Background:**

The purpose of this narrative review is to examine the literature investigating a causal relationship between stress and migraine and evaluate its implications for managing migraine.

**Methods:**

PubMed, PsycINFO and CINAHL were searched from 1988 to August 2021, identifying 2223 records evaluating the relationship between stress and migraine. Records were systematically screened. All potentially relevant records were thematically categorized into six mechanistic groups. Within each group the most recent reports providing new insights were cited.

**Results:**

First, studies have demonstrated an association of uncertain causality between high stress loads from stressful life events, daily hassles or other sources, and the incidence of new-onset migraine. Second, major stressful life events seem to precede the transformation from episodic to chronic migraine. Third, there is some evidence for changes in levels of stress as a risk factor for migraine attacks. Research also suggests there may be a reversed causality or that stress-trigger patterns are too individually heterogeneous for any generalized causality. Fourth, migraine symptom burden seems to increase in a setting of stress, partially driven by psychiatric comorbidity. Fifth, stress may induce sensitization and altered cortical excitability, partially explaining attack triggering, development of chronic migraine, and increased symptom burden including interictal symptom burden such as allodynia, photophobia or anxiety. Finally, behavioral interventions and forecasting models including stress variables seem to be useful in managing migraine.

**Conclusion:**

The exact causal relationships in which stress causes incidence, chronification, migraine attacks, or increased burden of migraine remains unclear. Several individuals benefit from stress-oriented therapies, and such therapies should be offered as an adjuvant to conventional treatment and to those with a preference. Further understanding the relationship between stress, migraine and effective therapeutic options is likely to be improved by characterizing individual patterns of stress and migraine, and may in turn improve therapeutics.

## Background

The definition of stress has varied through the ages and literature, but generally speaking, stress can be defined as an organism’s perception of and response to a perceived stressor. This includes how the body responds, both physiologically and psychosocially, to perceived threats, challenges, or physical or psychological barriers.

The physiological stress response involves the autonomic nervous system, especially the hypothalamic-pituitary-adrenal (HPA) axis, and several other structures which undergo neurobiological changes [1, 2]. Any stressor, both mental and/or physical, can elicit the physiological response resulting in pronounced sympathetic outflow and release of stress hormones such as epinephrine, norepinephrine, and cortisol. Increased activity of these stress systems induces behavioral, cardiovascular, endocrine, and metabolic cascades that enable the individual to fight, flee or freeze, to cope with the stress. The physiological stress response may be quantified in various ways, for example measuring the effector hormones (e.g., cortisol), or measuring the effect they produce (e.g., an increase in heart rate or blood pressure).

In addition to the physiological stress response, stress perception has a subjective side, i.e., how an individual perceives, reacts to, and copes with stress. This is described in the transactional stress model by Lazarus [3] which explains perceived stress as resulting in part from the “imbalance between demands and resources”, or a response where “pressure exceeds one’s perceived ability to cope.” Within this transactional model, stress ocurrs when perceived demands exceed perceived resources [4]. Perceived stress may be quantified using various patient reported outcome measures. A primary focus of cognitive behavioral therapy for migraine and related behavioral therapies involves teaching participants to first recognize the physiological and cognitive stress response, then observe their immediate automatic thoughts and then reappraise or reframe, replacing dysfunctional thoughts with more realistic thoughts [5]. Biofeedback and relaxation therapies also teach participants to recognize physiological and psychological activation and then engage in activities intended to reduce sympathetic activation and engage in mental and physical states of calm and well being [6].

The neuronal and hormonal changes associated with stress and the perceived psychological stress response may have multiple relationship to migraine. Stress may provoke the new-onset of migraine (incidence), it may act as a risk factor for migraine attacks, it may amplify migraine disability and/or burden; and it may contribute to the development of chronic migraine; Moreover, migraine attacks themselves and the resulting disability and impact might be a stressor in itself resulting in a vicious feedback cycle [7]. These relationships are exemplified in a common patient belief that “stress causes migraine.” [8] But, does the conventional belief hold? The aim of this article is to review currently available evidence and hypotheses for a causal relationship between stress and migraine and discuss important implications for management.

## Methods

To identify relevant literature for this narrative review, PubMed, PsycINFO and CINAHL databases were searched for available records from 1 January 1988—to include all relevant records after the inception of the International Classification of Headache Disorders 1st edition—and last updated on 10 August 2021. The following search term was used across all three databases: (“stress” AND “migraine”), identifying a total of 3124 records. 743 duplicates were identified automatically in the citation manager EndNote 20 (Clarivate, US). Another 158 duplicates were manually identified. The remaining 2223 records were screened based on title and abstract, identifying 208 records that were deemed relevant to appraise the casual relationship between stress and migraine or its implications for management. These 208 records were reviewed and thematically categorized [9]. Records not available online, not available in English, and commentaries were excluded. From the thematic categorization six mechanistic groups crystallized:

1. Is stress a predisposing factor for migraine disease onset (incidence)?
2. Is stress a predisposing factor for migraine chronification (new onset of chronic migraine)?
3. Is stress a risk factor for migraine attacks?
4. Is migraine-related disability and/or burden increased by stress?
5. What are the physiological correlates of stress and migraine?
6. What are the implications of stress for migraine management?

**Fig. 1:**
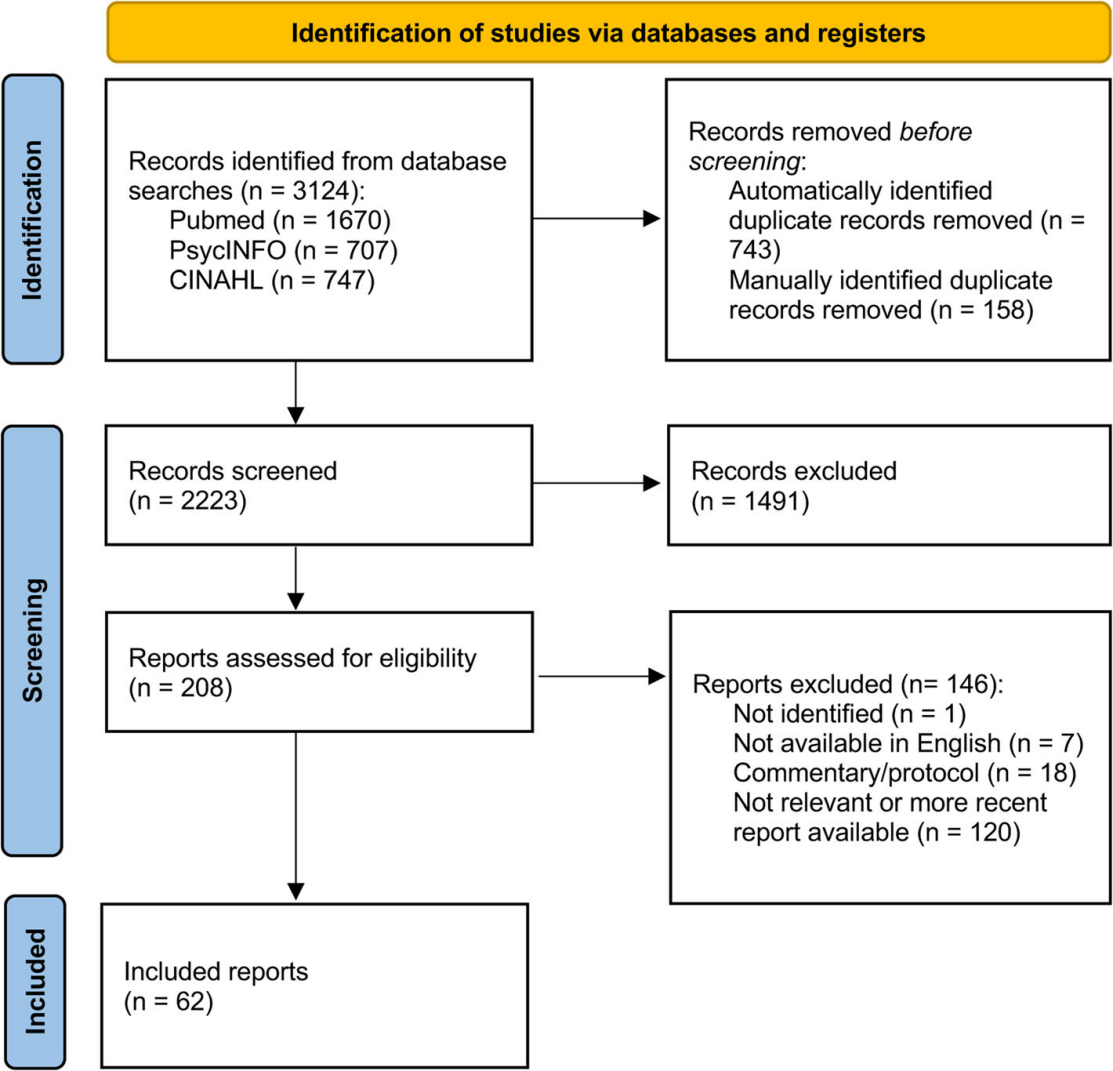
PRISMA flow diagram

Figure 1 is a PRISMA flowchart depicting the record selection process, and reasons for exclusion. 146 of the 208 identified records were excluded based upon the exclusion criteria. A total of 62 records from the electronic literature search are cited in this paper. Within each of the six themes, we cite records that provide insights into the causal relationship between stress and migraine, and attempt to use the most recently dated records.

## Results

### Evidence for a causal relationship between stress and migraine

#### Stress as a factor in new-onset migraine (incidence)

Stress, in a variety of fashions, seems to be associated with the new-onset of migraine. In a longitudinal analysis of almost 20,000 female employees with no history of migraine at study entry, no association between job strain and migraine was found at follow-up [10]. However, high effort-reward imbalance was associated with a slightly increased risk of migraine at follow-up. The proportion of new migraine cases attributable to high effort-reward imbalance was 6.2%. The association of high effort-reward imbalance to new-onset migraine was again confirmed in a 2020 prospective occupational health study [11]. Similarly, a study from 2019 investigated the association between early-life stressors and new-onset adolescent headache [12]. There was an influence of early life family-level factors on the prospective risk of developing migraine moderated through symptoms of depression and anxiety. Moreover, adverse childhood experiences have been demonstrated to have a stable association with an adult diagnosis of migraine [13–15], and the association seems to be mediated through neuroticism [16]. Pain as a neonate, defined as prescribed analgesics during stays at the neonatal intensive care unit, has also shown an association with an earlier onset of migraine [17]. All of these studies suggest that unfavorable allostatic load, contextualized as stress, may contribute to the new onset of migraine. Nevertheless, the associations are moderate, and a direct causal link to stress remains unclear.

#### Stress as a factor in migraine chronification or the new onset of chronic migraine

In a comprehensive systematic review from 2019, risk factors for the new onset of chronic migraine were assessed [18]. The main risk factors for chronification were increased headache day frequency, depression, and certain categories of acute medication overuse. However, major stressful life events were also identified as a risk factor. One of the studies included in the review reported more major stressful life events in the year before or the same year as the onset of chronic daily headache [19]. Similar results were also found in a study by De Benedittis and colleagues, where chronic primary headache patients reported significantly more stressful life events with negative impact in the year before chronic headache onset compared to headache-free controls [20]. More recently, a study investigating the effects of exposure to the 2011 Utøya adolescent summer camp mass shootings found an increased risk of persistent weekly and daily migraine in the months following the terror [21]. Together, this indicates that major stressful events can be associated with the new onset of chronic migraine, although it is unclear if such events directly cause the progression.

#### Stress as a migraine attack risk factor

Stress is perhaps the most commonly self-reported migraine attack trigger or risk factor [22, 23]. A prospective study of more than twelve hundred consecutive migraine patients found that 76% of patients reported perceived identifiable “triggers”, with stress being the most commonly reported (80%) [24]. Even though stress is reported as the most common self-identified trigger, the literature has produced conflicting findings as to whether stress (both static levels or change in stress) actually causes migraine attacks. One of the main problems is the difficulty of genuinely establishing the causal attack “triggers” [25]. Simply asking patients to recall their usual headache triggers/risk factors or premonitory features retrospectively is limited by recall bias and false attribution. Because different variations and combination of factors may raise the risk of a migraine attack, and these factors have different potencies as risks for a migraine attack both on an interindividual and intraindividual level, we will use the term risk factor rather than trigger to connotate the increased probably of a subsequent attack. Several prospective studies using daily paper or e-diaries report a temporal relationship between stress and migraine that is significant for headache days vs. non-headache days [26–30]. These studies indeed reduce the influence of recall bias and false attribution—yet, fully resolving the temporal association, and influence of static or changing stress levels, is complex.

**Fig. 2:**
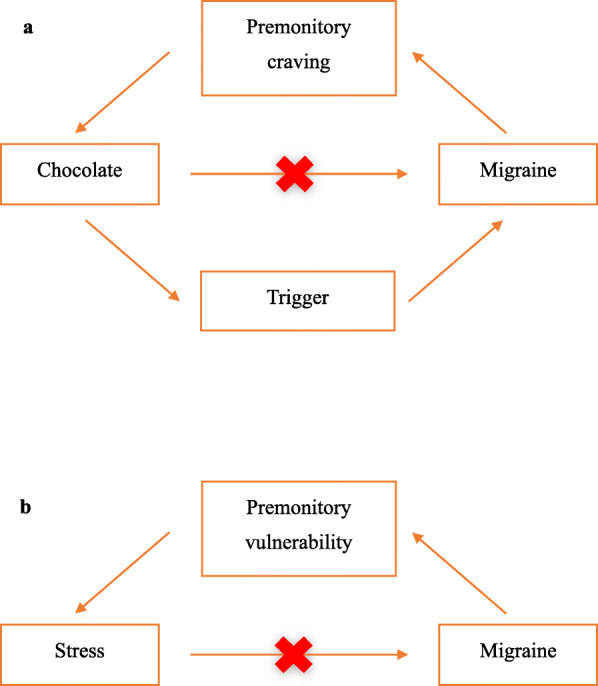
Directed acyclic graphs illustrating the causality between attack risk factors and migraine attacks. **a)** Recall the experiment evaluating if chocolate is a migraine trigger, or rather the case of reverse causality where the premonitory craving of an impending migraine attack elicits chocolate consumption. **b)** Similarly, the manifestation of stress preictally may be the case of a state of increased vulnerability to stressors

Let us ponder an example put forth by Lipton and colleagues: [25] Consider a patient that eats chocolate preictally. If chocolate consumption is a precipitant to the attack, then chocolate may be a risk factor. However, if the patient eats chocolate because she or he experiences cravings, the chocolate consumption could be a manifestation of the premonitory migraine phase (Fig. 2a). To distinguish these possibilities, a randomized trial with chocolate and placebo is required. In such a trial, one could administer chocolate or placebo to patients at a selected time and see if there is an increased probability of a migraine attack in the verum group (the group that received chocolate), which would support the notion of chocolate as a risk factor. Alternatively, patients could be randomized to chocolate or placebo when they crave it. If both the verum and the placebo groups have high but equal probabilities of headache, craving chocolate is a premonitory feature. If the verum group has a higher rate of migraine attacks, this suggests chocolate is a risk factor. Remarkably, variants of these studies have been conducted with various perceived triggers or risk factors including both chocolate and stress. In one study, a group of 27 patients with self-perceived triggers was exposed to their personal risk factors (flickering lights or strenuous exercise), and only 3 of 27 patients had a migraine attack after exposure, thus disfavoring the notion of causal risk factors [31]. Along the same lines, a 2021 study demonstrated that there was good agreement for reporting stress as an attack risk factor and experimental nitroglycerin-triggered premonitory mood change, favoring the notion of risk factors a manifestation of the premonitorium [32].

Turner and colleagues conducted a study to explore the conditions necessary to assign causal status to headache risk factors [33]. Based on the Neymar-Rubin causal effects model, the researchers identified three basic assumptions that need to be met to determine causality in headache risk factors: (1) constancy of the individual; (2) constancy of the risk factor effect; and (3) constancy of the risk factor presentation. Houle and Turner attempted to explore the assumption of constancy in risk factor presentation in a real-world natural experiment [34]. They concluded that it is challenging to find days with similar patterns of headache risk factors when relying on natural reliability. This means that the assumption of constancy in risk factors, and thereby causal inference, is likely violated in natural experiments. Thus, the validity of personal uncovering of risk factors is questioned.

A study from 2014 by Lipton and colleagues found that the decline of perceived stress was associated with the onset of migraine attacks. The study reported that static stress levels were not associated with subsequent migraine attacks, but decline in stress from one evening to the next was associated with increased migraine attack onset over the subsequent 6, 12, and 18 hours [26]. The authors offer several explanations as to why a reduction in stress was observed prior to migraine attacks. The findings may be explained by an unmeasured mediator, such as missed medication, skipped meals, or disturbed sleep, that arise as a consequence of stress, but may also be attributed to a reversed causality. During the premonitory phase, there may be a period of increased vulnerability to stress followed by a phase of decreased vulnerability to stress. This is akin to the example proposed earlier with chocolate, i.e., increased stress is a manifestation of an impending attack (Fig. 2b). To date, data are mixed on the “let down pattern” of reductions in stress triggering migraine attacks. Studies both support [35, 36] and disclaim the support of “weekend”, “honeymoon” and “let-down” migraines [28, 37].

However, two recent prospective diary studies adds additional insights [27, 38]. In the first study, a digital health platform was used to prospectively capture stress and headache data and evaluate if the patterns of perceived stress varied through the migraine cycle [38]. Perceived stress was rated once daily on a 0–10 scale. Days were categorized into pre-migraine days, migraine days, post-migraine days and interictal days. Using a cluster analysis, the researchers found three dominant patterns of perceived stress across the migraine cycle: “let down” pattern; “flat pattern”; and “stress as a trigger/symptom” pattern [38]. The majority of headache episodes were assigned to cluster 2 (flat). Approximately one-quarter were assigned to cluster 3 (trigger), and the rest was assigned to cluster 1 (let down). Interestingly, very few patients had more than 90% of their headache episodes in one cluster. No patients had > 90% of episodes in cluster 1 or 3, indicating that there were no exclusively “let-down” or “trigger” pattern patients. The second study suggests that changes in several key variables relative to an individual’s typical habits had the most power as risk factors [27]. Information was collected on daily levels of several commonly perceived attack risk factors including stress (Daily Stress Inventory), number of caffeinated and alcoholic beverages, and mood disturbances. The probability of observing variations in each trigger was used to estimate the “surprisal” of experiencing each trigger. When expressed as units of “surprisal”, the highest odds ratio for an attack was found for stress (1.30; 95% CI 1.14 to 1.46). Together, this illustrates a vast heterogeneity in the manifestation of stress as a migraine attack risk factor, not only between individuals, but also from attack to attack within individuals—hampering the establishment of any simple or generalized causal relationship between stress as a risk factor and migraine.

#### Migraine-related disability, burden, impact and frequency in relation to stress

Even though there is no definite evidence that stress incites the incidence or progression of migraine, or directly triggers migraine attacks, there is a general understanding of a worsening of migraine burden in the setting of stress. A large prospective study with more than 5000 participants found that stress intensity was associated with headaches for individuals with tension-type headache, migraine and, coexisting tension-type headache and migraine [39]. They found a 4.3% increase in headache days for each 10-point stress increase on a 0–100 visual analog scale. Correlation analyses were adjusted for sex, age, abortive drug consumption, drinking, smoking, body mass index, and education, meaning that higher levels of stress are associated with a higher migraine symptom burden. Similarly, several observational studies demonstrate an association between stress and migraine symptom burden. Higher migraine frequency is associated with higher levels of perceived stress [40]. High job strain, resulting in lack of time for personal care and leisure, is associated with an increased odds of migraine [41, 42]. Twenty-four hour shifts among medical residents increases migraine-related disability [43]. Among women, high levels of stress contribute to and increase the negative effects obesity has on migraine [44]. Stress facilitates the negative consequences poor sleep has on migraine occurrence [45, 46]. Stress is associated with poorer response to acute treatment in chronic migraine patients [47]. Interestingly, minor life events, or daily hassles, seems to be more associated with migraine-related disability than major life events [48].

On the other hand, a case-control study from 2017 with 227 patients found that episodic migraine patients had no more perceived stress than controls after adjusting for depression and anxiety [49]. This indicates that migraine itself does not necessarily result in perceived stress. Remarkably, the chronic migraine patients had significantly more stress after adjusting for depression and anxiety, suggesting that chronic migraine appears to be a specific factor for perceived stress. If the total migraine symptom burden affects levels of perceived stress, one should consider the option that the migraine—in itself—is an essential factor driving stress. In fact, several studies have shown that individuals with severe headaches appraise events more negatively, which may contribute to increased stress [50]. This plausible collateral causality between stress and migraine means that stress indeed is a part of a vicious reinforcing feedback cycle involving stress and migraine [51].

Finally, it is interesting to mention changes in migraine symptom burden during the SARS-CoV-2 lockdowns, viewed in light of the stress caused by the COVID-19 pandemic and isolation. Several recent studies have produced conflicting findings [52]. As an example, one survey from Italy found an overall reduction in headache frequency and intensity during the quarantine compared to pre-quarantine [53]. On the other hand, a study from Spain found worsening of the usual pain during lockdown [54]. It may be that the perception of the pandemic of the individual combined with individual circumstances, resources and demands may lead to profoundly different impacts for different individuals. Future reports summarizing these findings will likely provide a better understanding of how global pandemics, cautionary measures such as lockdowns and mask mandates and their repercussions affects the migraine population.

#### Physiological measures of stress and migraine

The studies mentioned above have chiefly assessed the relationship between perceived stress and migraine—but how does this correlate to physiological measures? Herein we will briefly review the physiological changes corresponding to stress and migraine.

Stress induces a series of neurophysiological changes that may aid in understanding the stress-related triggering of migraine attacks. In a laboratory study of knock-in S218L familial hemiplegic migraine mutation mice, relief after chronic stress led to a lower cortical spreading depression threshold [55]. This model may, in part, explain why some patients experience “let down” migraine. Another laboratory study found that patients who did not report stress as a risk factor displayed deficient habituation to visual evoked potentials, suggesting that stress-sensitive patients lack this altered cortical excitability threshold [56]. Moreover, recall the idea that migraine risk factors may be a case of reversed causality, e.g., identifying chocolate as a risk factor may be the manifestation of a premonitory craving. Neuroimaging studies demonstrate that stress may be a manifestation of premonitory activation of pain processing regions, including the hippocampus [57, 58], indicating that perceived stress is a manifestation of the impending attack.

Next, stress leads to central sensitization and hyperalgesia, which may concomitantly contribute to the debilitating characteristics of the migraine attack. Animal models show that both acute and chronic stress may enhance nociceptive responses [59]. Both peripheral and central sensitization seem to be important pathophysiological factors, contributing to many of the clinical characteristics of migraine including throbbing pain, motion sensitivity, hyperalgesia and allodynia [60] which may be exaggerated by stress. A recent study by Avona and colleagues [61] demonstrated that stress induced in mice produced a migraine-like state of hyperalgesia with increased response to the artificial migraine trigger sodium nitroprusside and subsequent blocking by calcitonin gene related peptide monoclonal antibodies. Similarly, allodynia exerts some effect on migraine-related disability, independent of the pain itself, and the relationship is partially driven by stress [62]. Moreover, emerging research has elucidated the role of NMDA receptors and microRNA in stress-related conditions such as major depressive disorder. Accumulated research suggests that chronic stress leads to an upregulation of NMDA receptor activation [2]. Glutamatergic medications acting as NMDA antagonists have shown an antidepressant and stabilizing effect in several brain circuits in animal models. Likewise, at the molecular level, dysregulation of microRNA seems to play a role in both acute and chronic stress and neuropsychiatric and affective disorders associated with stress [63]. It is conceivable that similar mechanisms apply to migraine in which stress seems to play a role.

Finally, the autonomic nervous system and HPA axis is worth mentioning. A body of research has traditionally focused on cardiovascular alterations among individuals with migraine [64], but there is now a trend toward the interactions of migraine and stress on the autonomic nervous system and HPA axis function. Yet, there are conflicting findings. Both lower and higher baseline sympathetic tone have been demonstrated in migraine patients [65–67]. Migraine patients also display significantly higher pain responses and sympathetic activation to stress than controls [68, 69]. In total, there seems to be a sympathetic impairment interictally with increased sympathetic responsiveness during the migraine attack [70]. One possible explanation of this phenomenon might be that the constant stress by repeated headaches results in a sympathetic downregulation with a *paradoxical* hyperactivation during subsequent headaches. Studies of HPA activation have also produced conflicting findings. A systematic review found conflicting findings on the levels of corticotropic hormones in migraine patients, and the authors concluded that corticotropic hormones, at least as a direct causal link, seem to be irrelevant in the pathophysiology of migraine [71].

### Implications for management

#### Behavioral interventions

Behavioral treatments with evidence for migraine prevention include stress management, relaxation therapy, cognitive behavioral therapy, biofeedback, mindfulness-based therapies and acceptance and commitment therapy [72–76]. The US headache consortium has given relaxation therapy, cognitive behavioral therapy, and biofeedback Grade A evidence for migraine prevention [77]. Most, if not all, of these behavioral treatments include improvements in stress management as well as adjusting expectations and perceptions as therapeutic targets. However, in many of the studies of behavioral interventions, it has been illustrated that the reduction in headache frequency is not a function of the intended beneficial target organ response [51, 78–80]. For example, in biofeedback training, improvement of circulation through hand warming does not necessarily translate to improvements in migraine outcomes. Thus, improvement in migraine and headache outcomes may be a consequence of non-specific effects, self-efficacy and the learned ability to cope with stressors combined with benefits of relaxation training to the nervous system—rather than stress reduction *alone* [80]. Moreover, trials of behavioral interventions have methodological challenges when using traditional pharmacological clinical trial standards, such as managing blinding and creating ideal control groups, which further hamper establishing causal effects. Even though the exact causal mechanisms of how stress management leads to improved migraine symptomatology are unclear, stress is a negative prognostic factor for migraine management and quality of life, clinical trial data and real-world experience show that many individuals have preventive benefits from established therapies, and some individuals prefer behavioral interventions. Therefore attention to individual management of stress is warranted and important [72, 77, 81].

A long-standing tradition in migraine management has been to advise patients to avoid headache risk factors, including increased stress. This strategy is traditionally implemented alongside general lifestyle measures such as good sleep hygiene, routine meal schedules, and regular exercise. Even though specific programs for identifying risk factors have been developed [82], some researchers suggest that complete avoidance of risk factors may have detrimental effects, in part driven by trigger sensitization [83, 84]. From this, the new behavioral management strategy termed “Learning to Cope with Triggers” emerged [85]. Therapist and patient would identify triggers and decide what strategy to use with them. Three main strategies were employed: exposure to find out if the trigger resulted in a migraine attack; exposure to achieve desensitization/habituation; and exposure to enable practicing coping skills. A randomized controlled trial with 127 participants found that “Learning to Cope with Triggers” was superior to both waiting-list and avoidance for the outcomes headache frequency and abortive drug consumption [85]. Of note, the study merged both migraine and tension-type headache.

One promising behavioral intervention that directly targets stress management and perception is mindfulness-based stress reduction (MBSR). A meta-analysis from 2018, including five small sample studies of migraine and tension-type headache, found no benefit for MBSR in treating chronic headache [86]. In more recent randomized controlled trials, MBSR has been found to be superior to simple stress management with regards to headache frequency reduction [87]; superior to headache education with regards to reduction in disability and improvements in quality of life [88]; and superior to progressive muscle relaxation with regards to adaptive coping strategies, self-efficacy and reduced pain perception [89]. Of note, the two latter studies failed the primary clinical outcome of improvement in migraine frequency. In addition to the MBSR studies mentioned above, mindfulness-based cognitive therapy for migraine has demonstrated efficacy to reduce headache-related disability and attack level disability, and is another promising emerging treatment option [74]. Overall, there is no unambiguous evidence that mindfulness-based therapies is better than traditional guideline recommended behavioral interventions for migraine prevention and management in terms of headache day reduction; however, it is a promising emerging treatment for addressing migraine-related disability as well as other important outcomes.

#### Pharmacological interventions

Although pharmacotherapy alone is improbable to be paramount in managing migraine in a context of stress, behavioral interventions can be combined with pharmacotherapy to enhance the effects. Combining drugs and behavioral treatment seems to be more effective than betablockers alone or behavioral treatment alone [90]. In a four-armed randomized controlled trial, 232 migraine patients were randomized to placebo, betablockers, behavioral treatment and placebo or behavioral treatment and betablocker [90]. The combination of betablockers and behavioral treatments improved treatment outcomes and outcomes of acute treatment. The same benefits of combining drugs and behavioral treatments has been observed in a trial children and adolescents, in which amitriptyline plus cognitive behavioral therapy was superior to amitriptyline alone [91]. The study did not evaluate cognitive behavioral therapy alone, or placebo alone, making it unclear if it’s the behavioral therapy or the medications that is most efficient in the pediatric population.

#### Predictive models

A promising and emerging topic of research is the use of risk factor and premonitory features, including stress, in predictive models and forecasting models [92]. One very promising study by Houle and colleagues [93] looked into predicting headaches based on self-reported perceived stress. To the best of our knowledge, this is the first study investigating if self-reported stress may be used to forecast headaches. In the study, a low-dimensional model using today’s level of stress combined with the presence or absence of headache could predict headache tomorrow with an out-of-sample area under the curve precision of 0.65. Such predictive models can likely further improve the management of migraine for several reasons. Reducing the unpredictability of attacks might, in turn, lead to breaking the vicious circle of stress-migraine-stress. Predicting attacks increases self-efficacy among patients and allows for tailored stress management and management of factors that indeed predict attacks. Forecasting enables so-called preemptive treatment (or mini-prophylaxis) in which medication is focused on days with an increased probability of attacks. However, preemptive drug treatment based on forecasting models should only be utilized if future predictive models are highly accurate due to the increased risk of medication overuse in patients with higher frequency migraine. Another recent study examined changes in several potential risk factors to forecast migraine attacks [94]. In the study, a decrease in caffeine consumption, higher self-predicted probability of headache, and a higher level of stress were observed within the two days preceding a migraine attack. However, the multivariable model predicted migraine risk only slightly better than chance with a within-person C-statistic of 0.56. Both studies emphasize that the use of an electronic diary to capture data is effective but results in missed data and measurement errors which may compromise the validity and precision of the forecasting models. Still, we are likely far from the plausible biological roof for forecasting migraine attacks. If there are underlying neurobiological differences that explain the vast heterogeneity of observed risk factors and premonitory factors, including stress, identifying these, and incorporating them into predictive models could result in high forecasting accuracy.

## Conclusion

Undoubtedly, there is a relationship between stress and migraine. The exact causal relationships in which stress causes incidence, chronification, migraine attacks, or increased burden of migraine remains unclear. Likely, the underlying neurobiology and pathophysiology is too complex, and the phenotype is too finely granulated to enable a generalized causal framework of migraine pathophysiology and treatment based on unitary stress biomarkers. However, actively engaging in how stress patterns unfold in each migraine patient could help the clinician in understanding why some patients transform to chronic migraine, how stress contributes as an attack risk factor, how stress may increase migraine symptom burden, and how to choose optimal therapy. Several individuals seem to benefit from stress management-oriented behavioral therapies, and such therapies should be offered as a adjuvant to pharmacological treatment or to those with a preference. Future studies should characterize large migraine cohorts to identify and exploit individual differences in treatment responsiveness of stress management-oriented therapies; and utilize all available data including triggers/ risk factors, premonitory features, and detailed facets of the physiological and psychosocial stress response, to create high-validity forecasting models.

## Data Availability

The literature database is available upon reasonable request to the authors.

## Abbreviations

HPA: Hypothalamic-pituitary-adrenal
MBSR: Mindfulness-based stress reduction
